# The Clinical Significance of Phosphorylated Heat Shock Protein 27 (HSPB1) in Pancreatic Cancer

**DOI:** 10.3390/ijms17010137

**Published:** 2016-01-21

**Authors:** Mitsuru Okuno, Seiji Adachi, Osamu Kozawa, Masahito Shimizu, Ichiro Yasuda

**Affiliations:** 1 Department of Gastroenterology, Gifu University Graduate School of Medicine, 1-1 Yanagido, Gifu 501-1194, Japan; mkobdkl@yahoo.co.jp (M.O.); shimim-gif@umin.ac.jp (M.S.); yasudaich@gmail.com (I.Y.); 2 Department of Pharmacology, Gifu University Graduate School of Medicine, 1-1 Yanagido, Gifu 501-1194, Japan; okozawa@gifu-u.ac.jp

**Keywords:** HSP27, pancreatic cancer, phosphorylated HSP27, chemosensitivity, prognosis

## Abstract

Pancreatic cancer is one of most aggressive forms of cancer. After clinical detection it exhibits fast metastatic growth. Heat shock protein 27 (HSP27; HSPB1) has been characterized as a molecular chaperone which modifies the structures and functions of other proteins in cells when they are exposed to various stresses, such as chemotherapy. While the administration of gemcitabine, an anti-tumor drug, has been the standard treatment for patients with advanced pancreatic cancer, accumulating evidence shows that HSP27 plays a key role in the chemosensitivity to gemcitabine. In addition, phosphorylated HSP27 induced by gemcitabine has been associated with the inhibition of pancreatic cancer cell growth. In this review, we summarize the role of phosphorylated HSP27, as well as HSP27, in the regulation of chemosensitivity in pancreatic cancer.

## 1. Introduction

Pancreatic cancer, which causes approximately 266,000 deaths per year, is the eighth leading cause of cancer-related deaths worldwide [1]. Surgery remains the best chance for achieving a cure, but at the time of diagnosis only approximately 10% of patients are eligible for surgery [2]. The majority of patients (80%–90%) present with incurable metastatic or locally-advanced disease. Therefore, the survival of patients with pancreatic cancer largely depends on chemotherapy.

Gemcitabine is a nucleoside analog of deoxycytidine which is enzymatically activated inside the cell where it subsequently inhibits DNA synthesis and induces apoptosis [3]. Thus far, gemcitabine has been shown to improve median overall survival from 4.4 to 5.6 months, in comparison to fluorouracil (5-FU), which is the chemotherapy that was classically used in the treatment of pancreatic cancer [4]. In 2011, Conroy *et al.*, reported the efficacy of FOLFIRINOX, a combination therapy that is used in the treatment of metastatic pancreatic cancer which consists of a biweekly regimen of oxaliplatin, irinotecan, fluorouracil, and leucovorin [5]. They demonstrated that FOLFIRINOX was associated with a median overall survival improvement of more than 10 months in comparison to gemcitabine [5]. However, hematologic and non-hematologic events including neutropenia, febrile neutropenia, diarrhea, and sensory neuropathy are more frequently associated with the administration of FOLFIRINOX than with gemcitabine. In 2013, Von Hoff *et al.*, reported that nab-paclitaxel with gemcitabine was more effective than gemcitabine alone, and that the combination resulted in an improvement in median overall survival that ranged from 6.7 to 8.5 months [6]. The combination of nab-paclitaxel with gemcitabine was associated with a lower risk of adverse events than FOLFIRINOX. Thus, in the treatment of pancreatic cancer, gemcitabine remains an efficient anticancer drug. Moreover, a large number of potential makers have been identified in pancreatic cancer, but their clinical utility as prognostic tools remains under investigation. With regard to gemcitabine, human equilibrative nucleoside transporter 1 (hENT1) [7,8], ribonucleotide reductase M1 (RRM1) [9], B7H4 [10], DJ-1 [10], and heat shock protein 27 (HSP27; HSPB1) are considered to be prognostic markers of resistance to chemotherapy in pancreatic cancer.

HSPs were first discovered as stress-inducible proteins [11]. HSPs have been characterized as molecular chaperones which prevent aggregation of proteins [12]. Based on their molecular masses, HSPs are currently classified into seven families, including HSPA (HSP70), HSPB (small HSPs), HSPC (HSP90), and HSPH (HSP110) [13]. The high molecular weight HSPs, such as HSPA (HSP70) and HSPC (HSP90), act as molecular chaperones in protein folding, oligomerization, and translocation. Although the functions of low molecular weight HSPs are not well characterized as those of high molecular weight HSPs, it is recognized that they may have chaperone activities. Among small HSPs with monomer molecular mass in the range of 12–43 kDa, HSP27, an ATP-independent molecular chaperone, is induced by the heat shock associated with physical and chemical stresses, including radiation, oxidative stress, and various chemotherapies [11,13]. HSP27 is able to bind to improperly folded proteins and further transfer them to the ATP-dependent chaperones such as HSPA (HSP70) or to the protein degradation machines including proteasomes or autophagosomes. HSP27 can also interact with various components including the regulated programmed cell death machinery, upstream and downstream of mitochondrial events [13,14]. The functions of HSP27 are regulated by post-translational modifications such as phosphorylation [15,16]. Human HSP27 is phosphorylated mainly at three sites (Ser-15, Ser-78, and Ser-82), and the phosphorylation is catalyzed by various protein kinases including mitogen-activated protein (MAP) kinase activated protein kinase 2 (MAPKAPK-2) [17]. Unphosphorylated HSP27 forms large aggregated large oligomers while its phosphorylation results in the conformational changes, leading to dissociated small oligomers [16].

With regard to cancer, HSP27 appears to be constitutively expressed at high levels in various tumors, including lung [18], gastric [19], prostate [20], and pancreatic cancers [21]. HSP27 expression has been reported to be associated with a superior treatment response [22], prognosis [23], and tumor progression [24]. Phosphorylated HSP27 expression also has been reported to play a suppressive role in the cell growth [25,26], chemosensitivity [27,28,29]. In pancreatic cancer, the expression of HSP27 and the phosphorylated HSP27 state are considered to play a critical role in gemcitabine resistance [10,28,29,30,31,32,33,34].

## 2. How Does HSP27 Predict Chemosensitivity?

HSP27 mainly acts as a molecular chaperone in cells exposed to different stresses, including heat shock and chemotherapy. It consecutively counteracts the formation of misfolded proteins and allows for correct protein folding [35,36]. In addition to these functions, HSP27 has been implicated in proteasome-mediated protein degradation as well as the regulation of the apoptotic pathway [13]. However, its precise role is still not fully understood.

Increasing evidence shows that HSP27 may predict the response of tumors in individual patients to radio- and chemotherapy. Studies have investigated the response in bladder [37], breast [38], esophageal [39], ovarian [40], and prostate cancers [41]. However, the role of HSP27 as a predictive marker for chemosensitivity in pancreatic cancer remains to be elucidated. There are several reports regarding indirect evidence on the influence of HSP27 on chemotherapy, including gemcitabine. Schafer *et al.*, [32] reported that HSP27 was an independent prognostic marker and that the better survival demonstrated in patients with HSP27-positive tumors may be partly attributable to a better response to gemcitabine. Additionally, Guo *et al.*, [33] reported that the overexpression of HSP27 increases the sensitivity to gemcitabine in the pancreatic cancer cell line, PL5, which is consistent with the *in vivo* results shown by Schafer *et al*. By contrast, Liu *et al.*, [30] reported that the expression of HSP27 was decreased in a gemcitabine-resistant pancreatic cancer cell line, Capan-1. Taken together, a high expression of HSP27 in pancreatic cancer cells appears to lead to high sensitivity to gemcitabine.

On the contrary, it has been reported that HSP27 plays a role in resistance to gemcitabine treatment or that it causes gemcitabine treatment to become irrelevant. Mori-Iwamoto *et al.*, [31] reported that the low expression of HSP27 is related to a better rate of survival in patients with pancreatic cancer in an *in vivo* study that used specimens obtained by endoscopic ultrasound-guided fine-needle aspiration (EUS-FNA). This article also reported that knockdown of the HSP27 expression using siRNA targeting HSP27 increased gemcitabine sensitivity even in the gemcitabine-resistant pancreatic cell line, KLM1-R. Similarly, Taba *et al.*, [42] reported that gemcitabine-resistant cell lines, KLM1-R and PK59, exhibited increased gemcitabine sensitivity after inhibition of the HSP27 expression by KNK43, a known HSP inhibitor that causes a decreased HSP27 expression in these cells. Zhang *et al.*, [43] also reported that SW1900/GEM, a gemcitabine-resistant cell line, exhibited increased gemcitabine sensitivity when the HSP27 expression was inhibited by shRNA specific for HSP27. Similar results were also shown in MiaPaCa-2, HPAC, and BxPC3 cells [34]. Additionally, a high expression HSP27 pancreatic cell line, MiaPaCa-2-HSP27, was resistant to gemcitabine-induced apoptosis compared with a low expression HSP27 cell line, MiaPaCa-2-Mock [21]. These studies suggest that the HSP27 expression is related to gemcitabine resistance. In our previous study, the HSP27 expression level did not affect the cell growth or sensitivity to gemcitabine in HSP27-transfected Panc1 pancreatic cell lines [29]. Moreover, there was no relationship between the HSP27 expression and gemcitabine sensitivity in another *in vivo* study [10], which was inconsistent with the previous studies showing that a low expression of HSP27 results in a better survival [31] or that HSP27-positive tumors were an independent prognostic marker [32] (Table 1). Taken together, the relationship between the HSP27 expression and sensitivity to gemcitabine differs according to the cell line used; therefore, the effect of the HSP27 expression on gemcitabine sensitivity must be investigated using various pancreatic cancer cell lines under the same conditions.

**Table 1:** The relationship between the HSP27 expression and the response to gemcitabine.

Author	Year	*in Vitro/Vivo*	HSP27 Expression	Response to Gemcitabine	Pancreatic Cancer Cell Lines (Response to Gemcitabine)
Mori-Iwamoto *et al.* [31]	2007	*in vivo/vitro*	↑	↓	KLM1-R (resistant)
Taba *et al.* [42]	2011	*in vitro*	↓	↑	KLM1-R, PK59 (resistant)
Baylot *et al.* [21]	2011	*in vitro*	↑	↓	MiaPaCa-2 (resistant)
Nakashima *et al.* [29]	2011	*in vitro*	not related	not related	Panc1
Liu *et al.* [30]	2012	*in vitro*	↓	↓	Capan-1
Schafer *et al.* [32]	2012	*in vivo/vitro*	↑	↑	PL5
Tsiaousidou *et al.* [10]	2013	*in vivo*	not related	not related	–
Guo *et al.* [33]	2014	*in vitro*	↑	↑	PL5
Kang *et al.* [34]	2015	*in vitro*	↓	↑	MiaPaCa-2, HPAC, BxPC3
Zhang *et al.* [43]	2015	*in vitro*	↓	↑	SW1900, SW1900/GEM (resistant)

As for paclitaxel, the role of HSP27 on the response to paclitaxel in cervical [44], ovarian [45,46], prostate [47], and bladder cancer [48] were reported recently. The cervical cancer tissues which were sensitive to paclitaxel or cisplatin showed the decreased expression of HSP27 after chemotherapy [44]. In addition, down-regulation of HSP27 expression increased the chemosensitivity to paclitaxel and paclitaxel-induced apoptosis in ovarian cancer [45]. Moreover, the expression of HSP27 helps progression by inhibiting apoptotic cell death in the bladder cancer patients treated with paclitaxel [48]. Taken together, it is more likely that higher HSP27 expression plays a protective role in cell proliferation of various cancers with paclitaxel-based chemotherapy. Future studies using a molecular approach, as well as large cohort study of patients treated with gemcitabine or paclitaxel, will be required to elucidate the mechanisms underlying the role of HSP27 as a prognostic marker.

## 3. How Does Phosphorylated HSP27 Affect Chemosensitivity?

Phosphorylated HSP27 reportedly plays a suppressive role in the cell growth of hepatocellular carcinomas [25,26]. In contrast, Matsunaga *et al.*, showed that the inhibition of HSP27 phosphorylation results in the increased sensitivity of colorectal cancer cells to chemotherapy (5-FU) [27]. However, the exact role of phosphorylated HSP27 in the various types of cancer is not fully understood.

With regard to pancreatic cancer, several reports discuss chemosensitivity to gemcitabine. Habiro *et al.*, reported that gemcitabine-induced apoptosis is mediated by p38 MAP kinase activation [49]. Taba *et al.* showed that the phosphorylation levels of HSP27 at Ser-78 and Ser-82 are elevated in gemcitabine-resistant pancreatic cancer cells, KLM1-R, in comparison to gemcitabine-sensitive pancreatic cancer cells, KLM1 [28].

**Figure 1:**
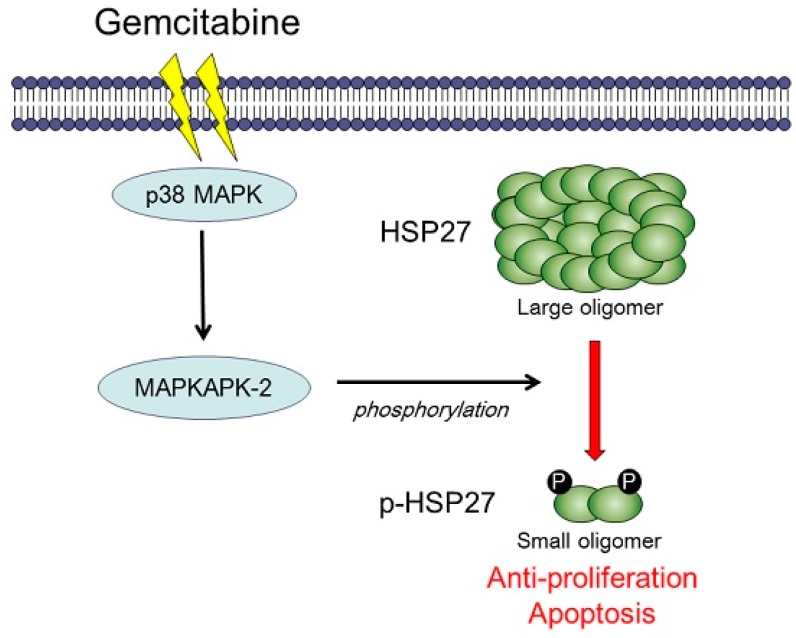
A schematic representation of the role of HSP27 in the sensitivity to gemcitabine in human pancreatic cancer. Gemcitabine induces the phosphorylation of HSP27 via the MAPK-MAPKAPK-2 pathway and phosphorylated HSP27 leads cells to growth suppression in pancreatic cancer.

On the contrary, Kang *et al.*, reported that the ratio of phosphorylated HSP27 to non-phosphorylated HSP27 was significantly increased after gemcitabine treatment using pancreatic cancer cell lines MiaPaCa-2, HPAC, and BxPC3, and that an increase in the ratio of phosphorylated HSP27 to non-phosphorylated HSP27 was related to cell death [34]. In our recent study [29], we investigated the relationship between the effect of gemcitabine and the phosphorylated HSP27 state using pancreatic cancer cell lines, KP3 and Panc1. We found that gemcitabine induces the phosphorylation of p38 MAPK, and subsequently MAPKAPK-2, which leads to the phosphorylation of HSP27 at Ser-15, Ser-78, and Ser-82 without affecting the total level of HSP27 [29]. Moreover, the phosphorylation of HSP27 induced by gemcitabine has a suppressive role in the pancreatic cancer cell growth and induces apoptosis [29]. In order to elucidate the precise role of phosphorylated HSP27 in the sensitivity to gemcitabine, we established two types of mutant HSP27-transfected Panc1 pancreatic cell lines, overexpressed non-phosphorylatable HSP27 cells and phosphorylated HSP27 cells [29], and found that the cell growth of phosphorylated HSP27 cells was markedly retarded compared with that of non-phosphorylatable HSP27 cells [29], indicating that gemcitabine-induced HSP27 phosphorylation via the p38 MAPK-MAPKAP-2 pathway leads to the growth suppression of pancreatic cancer cells. We summarized the hypothetical role of phosphorylated HSP27 in the gemcitabine-induced growth suppression of pancreatic cancer (Figure 1) and reviewed these studies in Table 2.

**Table 2:** The relationship between the phosphorylated HSP27 (p-HSP27) expression and the response to gemcitabine

Author	Year	*in Vitro/Vivo*	p-HSP27 Expression	Response to Gemcitabine	Pancreatic Cancer Cell Lines (Response to Gemcitabine)
Taba *et al.* [28]	2010	*in vitvo*	↑	↓	KLM1/KLM1-R (resistant)
Nakashima *et al.* [29]	2011	*in vitvo*	↑	↑	Panc1
Kang *et al.* [34]	2015	*in vitvo*	↑ (p-HSP27/HSP27)	↑	MiaPaCa-2, HPAC, BxPC3

## 4. Conclusions and Future Directions

Phosphorylated HSP27 could, therefore, be a potentially-useful biomarker that predicts the sensitivity of pancreatic cancer to gemcitabine-based chemotherapy. Further investigation might provide a more effective combination chemotherapy that uses gemcitabine in the treatment of human pancreatic cancer. Compounds which collaborate with HSP27 might, therefore, be useful in this regard.
